# Elimination of Viral Hepatitis in Low and Middle-Income Countries: Epidemiological Research Gaps

**DOI:** 10.1007/s40471-021-00273-6

**Published:** 2021-07-31

**Authors:** Antoine Jaquet, Guy Muula, Didier K. Ekouevi, Gilles Wandeler

**Affiliations:** 1 University of Bordeaux, Inserm, French National Research Institute for Sustainable Development (IRD), UMR, 1219 Bordeaux, France; 2 Centre for Infectious Disease Research in Zambia (CIDRZ), Lusaka, Zambia; 3 Département de santé publique, Faculté des Sciences de la santé, Faculté des Sciences de la santé, Université de Lomé, Lomé, Togo; 4 Programme PACCI, site ANRS, Abidjan, Côte d’Ivoire; 5 Department of Infectious Diseases, Bern University Hospital, Inselspital, University of Bern, 3010 Bern, Switzerland; 6 Institute of Social and Preventive Medicine, University of Bern, Bern, Switzerland

**Keywords:** Elimination, Viral hepatitis, Research gaps, LMIC, Sub-Saharan Africa

## Abstract

**Purpose of Review:**

The purpose of our review was to summarize current recommendations on testing strategies, antiviral therapy eligibility and monitoring, and prevention of mother-to-child transmission of chronic hepatitis B virus (HBV) and hepatitis C virus (HCV) infections, and to highlight major research gaps in low and middle-income countries (LMIC), with a particular focus on sub-Saharan Africa (SSA).

**Recent Findings:**

While data on the prevalence of HBV and HCV infections in LMIC are increasing, current knowledge on liver-related complications as well as on treatment outcomes remains limited. Furthermore, very little information is available on the feasibility and cost-effectiveness of large-scale testing and management strategies in high-prevalence settings. The availability of policy-relevant data is particularly scarce in SSA, which accounts for a significant part of the global burden of chronic viral hepatitis.

**Summary:**

Current recommendations on the management and monitoring of chronic viral hepatitis rely mainly on data from high-income settings. The global elimination of viral hepatitis will only be achieved if prevention, testing, and treatment strategies tailored to specific LMIC are implemented. In order to inform scalable and cost-effective interventions, dedicated research initiatives have to be undertaken. Future studies will have to include the evaluation of innovative testing strategies, the validation of simplified methods to diagnose liver cirrhosis and hepatocellular carcinoma, and the monitoring of long-term treatment outcomes and toxicity. In addition, national plans to achieve the elimination of HBV mother-to-child transmission are urgently needed, including effective ways to test pregnant women, treat those who are eligible, and ensure birth dose vaccination is given to all newborns.

## Introduction

Worldwide, chronic viral hepatitis infections cause over 1.3 million deaths per year, and account for half of liver cirrhosis and hepatocellular carcinoma (HCC) cases [1–3••]. Although chronic hepatitis B virus (HBV) and hepatitis C virus (HCV) infections affect 325 million people globally, fewer than 20% of them are aware of their diagnosis, and less than 10% have received appropriate antiviral therapy [3••]. These estimates are generally lowest in the most affected regions: for instance, the proportion of HBV infections diagnosed in 2015 was 0.3% in the African region and 2.3% in the Western Pacific region, which account together for two thirds of HBV infections globally.

In light of the increasing disease burden of chronic viral hepatitis, the World Health Assembly endorsed the Global Health Sector Strategy on viral hepatitis in 2016, and called for the elimination of viral hepatitis as a public health threat by 2030 [4]. The targets to be achieved by 2030 are ambitious: a 90% reduction in new cases and a 65% reduction in mortality due to chronic HBV and HCV infections [3••]. Both targets rely on the achievement of several specific objectives, including massive improvements in delivery of interventions to reduce HBV mother-to-child transmission (MTCT), and uptake of HBV and HCV testing and treatment. Reaching these objectives will depend on the successful implementation of evidence-based interventions, adapted to specific regions and populations. Still, many research gaps exist, especially regarding the epidemiology, testing strategies, antiviral therapy eligibility, monitoring of treated individuals and prevention of mother-to-child transmission of chronic viral hepatitis in low- and middle-income countries (LMIC). We aimed to summarize current recommendations on HBV and HCV testing and management, including those from the World Health Organization (WHO) and the European Association for the Study of the Liver (EASL) [5••–9], and highlight the main data gaps for LMIC (summarized in Table 1), with a focus on sub-Saharan Africa (SSA). Addressing these important research questions will be key in helping clinicians, policy makers, and funders pave the way towards elimination of viral hepatitis.

## Current Evidence and Research Gaps Across the Viral Hepatitis Care Cascade in Low and Middle-Income Countries

### Epidemiological Determinants and Testing Strategies

Detailed knowledge of the national and subnational HBV and HCV epidemiological determinants is key for the implementation of sound testing strategies.

#### HBV Infection

The prevalence and natural history of HBV infection in LMIC have been relatively well characterized over the last decades [10]. Based on the worldwide prevalence of HBsAg carriage, countries are considered to have low (<2%), intermediate (2–5%), or high (>5%) HBV prevalence. However, these national HBV estimates are likely to change over time as most LMIC have now implemented universal HBV vaccination in children as part of their expanded immunization program. Among the major indicators used by WHO to monitor progress towards elimination of HBV is the number of new infections in children under 5 years old. Empirical data on HBsAg carriage in this population is currently limited. While modeling approaches can contribute to our understanding of the burden of HBV infection and its dynamics, they will not replace serological surveys in this age category. In this specific population, a particular focus on infants exposed to HBV infection will be of interest to assess the efficacy of preventive interventions. In settings with an HBsAg prevalence ≥2% in the general population, focused testing in high risk populations alone will be insufficient to identify most individuals infected. Therefore, general population testing for HBV infection is recommended in many LMIC. This strategy implies the systematic testing of all adults regardless of their age or risk, and raises many challenges. First, data on the potential benefits of such an approach are very limited: A recent mathematical modeling study based on data from West Africa showed the cost-effectiveness of offering HBV testing and treatment to the general population, even if the prevalence was as low as 2% [11•]. Second, the implementation of such an approach raises many questions specific to LMIC, considering the limited resources allocated to healthcare and the absence of universal healthcare coverage. Therefore, providing HBV testing to the general population will have to rely on a combination of strategies, including provider-initiated and community-based testing.

#### HCV Infection

The epidemiology of HCV infection differs from HBV, with a more heterogeneous picture of HCV prevalence and transmission clusters, frequently linked to healthcare interventions (exposure to infected blood through unsafe injections) and risk behaviors (injection drug use) [12, 13]. The local specificities of the epidemiological determinants of HCV infection imply the need for more granular data on HCV prevalence to prioritize populations and geographic areas in need of particular attention. This is particularly true at a subnational level, where major regional variations are observed. For instance, in Burkina Faso, a national survey based on the 2010–2011 Demographic and Health Survey found a much higher HCV seroprevalence in the South-West region (13.2%; 95% CI: 10.6–15.7) compared to the national HCV seroprevalence (3.6%; 95% CI: 3.3–3.8) [14]. Another major limitation in current knowledge about HCV prevalence is the general lack of confirmation of active chronic HCV infection in most studies. High rates of false-positive HCV antibody tests have been reported in SSA, especially in the context of HIV coinfection [15]. In order to plan HCV management strategies and achieve appropriate linkage to care, countries need to rely on estimates of confirmed, active HCV infections, which implies the availability of affordable and accurate nucleic acid amplification tests. Given their reduced cost, point-of-care HCV core antigen tests may constitute a promising alternative to classical HCV polymerase chain reaction [16]. The prevalence of HCV infection being 2% in most LMIC, targeted strategies focused on sub-groups of the population with a higher risk of being infected are warranted.

#### Service Delivery of Testing for Viral Hepatitis in LMIC

In settings with weak healthcare systems, vertical health programs supported by external funders such as HIV/AIDS or Tuberculosis programs are particularly suited for systematic viral hepatitis testing and should set the example for more general testing. For instance, all persons living with HIV (PLWH) should be tested for HBV, given the need for HIV/HBV-coinfected individuals to receive tenofovir-containing antiretroviral therapy (ART). Despite the progress made in recent years, only a minority of PLWH are tested in clinical routine, as shown in a recent multi-cohort study from SSA [17]. According to WHO recommendations, HBV and HCV screening of blood donors should be mandatory, and linkage to care, counseling and treatment provided for those who test positive. While testing is the rule in blood banks throughout the world, notification and linkage to care of persons who test positive is rarely implemented in LMIC. Another unique opportunity to implement facility-based testing would be the antenatal clinic setting, albeit HBV testing, as opposed to HIV, is not free of charge. Failing to test pregnant women for HBV is a missed opportunity to prevent vertical transmission and to initiate antiviral therapy for women who need it for their own health. Universal testing will ultimately require broader provider-initiated testing approaches, including in general healthcare facilities, where knowledge of viral hepatitis management is often minimal. Surveys performed in SSA consistently highlighted the insufficient knowledge of viral hepatitis prevention, testing and treatment among medical practitioners as well as in the general population [18–21]. Eventually, only community-based testing strategies will allow reaching the majority of the general population, which does not have access to provider-based testing. Studies evaluating the implementation of innovative outreach initiatives, using rapid point-of-care tests and facilitated linkage to care, are urgently needed.

##### Important Research Gaps

1. How should HBV testing of the general population be implemented? Additional studies are needed to assess the benefit of universal HBV testing to inform health policy in countries already confronted to many additional health threats. Differentiated testing approaches prioritizing spouses, siblings, and household contacts of infected persons, as well as healthcare workers, should be evaluated. Such testing programs need to consider further diagnostic steps for HBsAg-positive individuals and evaluation of treatment eligibility in decentralized settings.
2. Is hepatitis delta infection a major threat in LMIC? Hepatitis delta causes the most severe form of viral hepatitis and increases the risk of liver-related mortality among HBV-infected persons [22]. Although large population-based studies have shown a prevalence of HDV infection reaching >50% of HBsAg-positive persons in some regions of Central Africa, estimates vary widely across settings and even within countries [23, 24]. A more detailed knowledge of the burden of HDV infection in LMIC is crucial to inform HBV care programs, as treatment options for HBV/HDV-coinfected individuals remain very limited.
3. Is birth cohort testing for HCV infection the best way forward? In many regions of the world, including SSA, a birth cohort effect on HCV prevalence has been shown; for instance, the HCV seroprevalence was found to increase dramatically with age in several areas of Cameroon, reaching 50% of among individuals born before 1950 [25]. As the cost-effectiveness of testing strategies focused on birth cohorts and high-risk groups has been established, efforts should be made to identify high prevalence populations across LMIC for intensified screening.

### Antiviral Therapy Eligibility and Modalities

The overall goals of antiviral therapy for viral hepatitis are the improvement of quality of life and the survival of infected persons by decreasing the risk of liver cirrhosis and HCC. Additional objectives are the prevention of transmission, for example from the mother to the child, as well as the reduction of symptoms related to extra-hepatic manifestations.

#### HBV Infection

The immediate goals of antiviral therapy for HBV infection are the suppression of HBV viral load and the normalization of liver transaminases [5••, 8]. Among HBeAg-positive individuals, conversion to HBeAgnegativity occurs in 30–40% after 5 years of antiviral therapy and leads to a phase of the infection with reduced activity (HBeAg-negative chronic HBV infection). Finally, HBsAg loss, also described as the functional cure of HBV infection, is currently seen as the ideal outcome of antiviral therapy, as it is associated with improvements in clinical outcomes, reduction of HCC incidence, and allows the cessation of antiviral therapy [26]. HBV functional cure generally occurs at a rate of 1% per person-year, but has been shown to be more likely among HIV/HBV-coinfected individuals on tenofovir-containing ART [27•, 28]. Many cases of active HBV infection will not require immediate antiviral therapy but should instead undergo strict monitoring, which consists of measurements of ALT, HBV DNA and liver fibrosis at regular intervals, depending on the phase of the infection. Table 2 summarizes current recommendations regarding eligibility for antiviral therapy in chronic HBV infection. In HIV/HBV co-infected individuals, tenofovir-containing ART should be initiated in all patients, regardless of CD4 cell count. The mainstay of antiviral therapy for HBV infection is the use of nucleoside analogues, including tenofovir disoproxil fumarate (TDF), tenofovir alafenamide (TAF), or entecavir, which lead to HBV suppression in >90% of cases.

#### HCV Infection

The immediate goal of antiviral therapy for HCV infection is to achieve a sustained virologic response, which is generally defined as the absence of HCV RNA in serum 12 weeks after completion (SVR12) [6••, 7]. Antiviral therapy for HCV infection should only be initiated in patients with HCV RNA-confirmed infection. Treatment is recommended for all patients with chronic HCV infection, except those with a short life expectancy that cannot be remediated by HCV therapy, liver transplantation, or another directed therapy. The use of new generation direct-acting antivirals (DAA) leads to SVR12 rates above 90% in treatment-naïve individuals, independent of HCV genotype or fibrosis stage. Two treatment regimens are favored as first-line therapy for DAA-naïve individuals without cirrhosis or compensated cirrhosis: glecaprevir/pibrentasvir for 8 weeks or sofosbuvir/velpatasvir for 12 weeks [7]. Drug–drug interactions remain a significant issue, especially in HIV-infected individuals treated with DAA: for instance, the prescription of sofosbuvir/velpatasvir or glecaprevir/pibrentasvir is contraindicated if efavirenz is part of the ART regimen.

##### Important Research Gaps

1. Should eligibility for antiviral therapy for HBV infection be more inclusive? Recent data have shown that potential pre-carcinogenetic activity starts early in HBeAg-positive individuals, despite the absence of overt liver damage [29]. In addition, little is known on the incidence of HCC among individuals infected during early childhood in SSA, where they are exposed to other carcinogens, such as aflatoxins. In the largest case series reported to date, the age at HCC diagnosis was shown to be lower in SSA than elsewhere [30•, 31]. Prospective studies investigating long-term liver-related complications of HBV infection are urgent to inform treatment eligibility guidelines.
2. Do we know enough about long-term toxicity of TDF and TAF? Although many studies have shown the association between TDF and renal tubular toxicity among PLWH [32], data from HBV-monoinfected individuals are scarce. The use of TAF seems beneficial for HIV/HBV-coinfected persons with renal dysfunction [33], but its potential association with metabolic complications makes it a questionable alternative [34]. More data are needed on toxicity of nucleoside analogues in HBV-infected persons, especially from regions where laboratory monitoring is difficult to implement.
3. Can the evaluation of HBV-related cirrhosis be done using serological scores? WHO recommends the use of the APRI score to diagnose liver cirrhosis in settings where transient elastography is not available [5••]. However, studies from large HBV cohorts in high-income countries as well as in Ethiopia confirmed the low diagnostic capacity of this score, which missed more than 50% of cases of cirrhosis [35•, 36]. Efforts to develop better non-invasive scores and to improve access to transient elastography or similar technologies are crucial.
4. Should genotype be determined before anti-HCV therapy? Recently, concerns have been raised on the efficacy of current DAA regimens in the presence of specific subgenotypes mainly present in SSA. For example, SVR12 rates were 60% among patients with sub-genotype 4r treated with sofosbuvir/ledipasvir in Rwanda [37•]. Data on treatment outcomes with the newest generation DAA combinations from SSA, including among individuals infected with unusual sub-genotypes, are eagerly awaited.
5. How can access to HCV therapy be improved? Although many LMIC are in the process of improving their access to generic drugs, prices of DAA remain too high for many countries, and many structural barriers impair the successful expansion of HCV therapy. Lessons from the global rollout of HIV therapy will be crucial to inform HCV treatment strategies

### Monitoring of Treated Individuals

In patients undergoing antiviral therapy, monitoring of treatment toxicity and success is important. The number and frequency of tests recommended have changed over time, especially in the context of the use of safe and potent DAA combinations for HCV. Table 3 shows the monitoring procedures currently recommended for patients eligible for antiviral therapy.

Although there is some consensus on the frequency of laboratory monitoring during and after antiviral therapy, many questions are still open.

#### Important Research Gaps

1. Which HBV-infected individuals to screen for HCC in the absence of cirrhosis? The poor prognosis of HCC when diagnosed late justifies surveillance using 6-monthly ultrasound examinations in patients at high risk. Besides the presence of liver cirrhosis, additional risk factors, including age and sex, predict HCC in HBV-infected individuals with or without HIV coinfection [38, 39•]. However, previous studies have not focused on African populations, where exposure to aflatoxin B1 may influence the risk of HCC. Given the structural barriers to HCC screening in LMIC, optimal risk stratification needs to be informed by prospective cohort studies with long-term follow-up.
2. When can antiviral therapy for HBV infection be stopped? In addition to patients with HBV functional cure, those with sustained HBeAg loss have also been considered for discontinuation of antiviral therapy [5••]. The potential for long-term toxicity of antiviral therapy needs to be balanced against the risk of hepatitis flare after its discontinuation. To date, no studies have evaluated the pros and cons of this strategy in SSA. The potential impact of treatment interruption strategies on clinical outcomes and costs in LMIC, as well as the necessary monitoring strategies after discontinuation, need to be evaluated in dedicated studies.
3. Should SVR be monitored after antiviral therapy for HCV infection? According to EASL, checking SVR may be dispensable in parts of the world with limited resources for health, given the high SVR12 rates expected with DAA-based regimens [7]. However, SVR monitoring may remain indicated for groups of patients with a high risk of reinfection or treatment failure. Future research should help identify groups of patients for which close monitoring is warranted in specific settings.

### Prevention of Mother-to-Child Transmission

In LMIC, HBV is typically transmitted from the infected mother to the child during birth or through household contact during early childhood. Based on existing recommendations on immunization from the WHO position paper in 2017, all infants should receive their first dose of hepatitis B vaccine within 24 h after birth, followed by two or three doses to complete the primary series [3••]. Delivery of hepatitis B birth dose vaccination should be a performance indicator for all immunization programs, and reporting and monitoring systems should be strengthened to improve data quality. However, only few LMIC have effectively introduced HBV birth dose vaccination so far [40]: In SSA, where only 50% of babies are delivered by a skilled birth attendant, coverage of birth dose is estimated by WHO to be <10% [41]. According to a meta-analysis including 31 studies from SSA published before March 2017, the pooled coverage rates of HBV birth dose was 1.3% (0.0–4.5) [42].

According to the recently published WHO guidelines on HBV antiviral prophylaxis in pregnancy, women testing positive for HBV infection with an HBV DNA ≥ 5.3 log10 IU/mL (≥ 200,000 IU/mL) should receive TDF prophylaxis from the 28th week of pregnancy to prevent MTCT [43••]. In settings in which antenatal HBV DNA testing is not available, HBeAg testing can be used as an alternative to HBV DNA to determine eligibility for antiviral prophylaxis. However, estimates of the effectiveness of testing and antiviral prophylaxis of pregnant women, in addition to HBV birth dose vaccination, essentially rely on data from Asia, where the determinants of HBV transmission are different from SSA.

#### Important Research Gaps

1. Which service delivery model is most effective for the provision of integrated HIV, syphilis, and viral hepatitis testing and care for pregnant women? Improving testing and treatment of these conditions in prenatal care is urgent, but not enough. A significant proportion of births occur outside of healthcare services and many women do not have access to antenatal care. Thus, innovative models of decentralized testing and birth dose vaccination considering local specificities and structural barriers will have to be evaluated.
2. Should TDF be prescribed regardless of HBV DNA level to prevent HBV MTCT if timely birth dose vaccination is not available? In some settings, antiviral prophylaxis is prescribed to prevent MTCT based on a single positive HBsAg test, in the absence of HBV viral load assessments. The efficacy and safety of this pragmatic approach should be evaluated. Conversely, if treatment eligibility has to be based on the level of HBV replication, POC HBeAg tests and/or DBS-based viral load testing need to be urgently validated.
3. Should antiviral prophylaxis be continued after postpartum? The incidence of hepatitis flares after the interruption of TDF prophylaxis is largely unknown, and women in LMIC are likely to experience subsequent pregnancies with the need to start antiviral prophylaxis during these pregnancies. In analogy to pragmatic decisions taken to address HIV MTCT in SSA [44], the feasibility and efficacy of continuous antiviral prophylaxis for women in childbearing age need to be assessed in large-scale studies.

## Conclusions

Despite recent advances in HBV and HCV management over the past decade, our review highlights the many challenges on the road to viral hepatitis elimination as a public health threat by 2030 in LMIC. Reducing viral hepatitis mortality by 65% globally will not be possible unless major improvements occur throughout the cascade of care. Importantly, countries should focus on achieving viral hepatitis elimination targets with their own service coverage initiatives that will have the maximum impact [45]. Provider-initiated and community-based strategies will have to be implemented in most LMIC to reach general population HBV testing, whereas HCV testing strategies will have to focus on most affected populations and consider birth cohorts. Dedicated research initiatives will have to evaluate affordable and scalable diagnostic procedures to detect liver fibrosis and screen for HCC in decentralized settings. Widespread access to antiviral therapy for eligible HBV-infected and HCV-infected individuals has to be guaranteed, and the capacity for the systematic monitoring of its efficacy and safety improved. Finally, there will be no elimination of HBV in LMIC without the prevention of MTCT: HBV testing of all pregnant women, as well as antiviral prophylaxis for those with high viral loads, and HBV birth dose vaccination for all newborns should be priorities for all countries [46].

To help address the challenges ahead, it will be crucial to learn the lessons from the fight against HIV in settings where the implementation of testing and treatment is challenging [47]. The tools to prevent, diagnose, and treat viral hepatitis are known and available. However, reaching the ambitious objectives set for elimination will depend on the strong commitment from the international community as well as from governments of the most affected countries to support the scale-up of key interventions. Current guidelines on viral hepatitis treatment and monitoring have been mostly based on data from high-income countries, where healthcare systems and the natural history of chronic viral hepatitis infections are different from SSA. Well-designed implementation science and clinical research studies from LMIC will be key in closing epidemiological data gaps and informing innovative and cost-effective strategies to be implemented at a large scale.

## Figures and Tables

**Table 1:** Summary table of important research gaps

**Epidemiological determinants and testing strategies**	How should HBV testing of the general population be implemented? Is hepatitis delta infection a major threat in LMIC? Is birth cohort testing for HCV infection the best way forward?
**Antiviral therapy eligibility and modalities**	Should eligibility for antiviral therapy for HBV infection be more inclusive? Do we know enough about long-term toxicity of TDF and TAF? Can the evaluation of HBV-related cirrhosis be done using serological scores? Should genotype be determined before anti-HCV therapy? How can access to HCV therapy be improved?
**Monitoring of treated individuals**	Which HBV-infected individuals to screen for HCC in the absence of cirrhosis? When can antiviral therapy for HBV infection be stopped? Should SVR be monitored after antiviral therapy for HCV infection?
**Prevention of mother-to-child transmission**	Which service delivery model is most effective for the provision of integrated HIV, syphilis and hepatitis B testing and care for pregnant women? Should TDF be prescribed regardless of HBV DNA level to prevent HBV MTCT if timely birth dose vaccination is not available? Should antiviral prophylaxis be continued after postpartum?

**Table 2:** Eligibility criteria for antiviral therapy in chronic HBV infection

Treatment eligibility	HBV viral load unavailable (WHO criteria)	HBV viral load available (EASL criteria)
Must be treated	- Presence of cirrhosis (clinical signs or based on non-invasive measurement) regardless of ALT level	- Presence of chronic hepatitis B* - Presence of cirrhosis and detectable HBV VL, regardless of ALT level. - Presence of HBV VL>2,000 IU/mL and ALT level >2ULN, regardless of fibrosis
May be treated	- Presence of chronic hepatitis B without cirrhosis but persistently elevated ALT and age >30 years (in particular)	- Presence of chronic HBeAg-positive HBV infection* and age >30 years, regardless of fibrosis - Presence of chronic HBV infection with family history of HCC or cirrhosis, or with extrahepatic manifestations

* Chronic hepatitis B defined by HBV DNA >2,000 IU/ml, ALT >ULN, and/or at least moderate liver necro-inflammation or fibrosis. Chronic HBV infection defined by persistently normal ALT and high HBV DNA levels

**Table 3:** Monitoring of patients on antiviral therapy for HBV and HCV infections (adapted from WHO and European guidelines [5–8])

	HBV	HCV
Laboratory		
Transaminases	3-monthly during year 1, 6-monthly thereafter	At treatment start (3 months after the end of therapy)
Renal function	3-monthly during year 1, 6-monthly thereafter	At treatment start and monthly during sofosbuvir treatment if renal dysfunction at baseline
Viral load	3-monthly during year 1, 6–12-monthly thereafter	Before treatment and 3 months after end of therapy (SVR12)
Serology	HBsAg yearly	Before treatment
Liver imaging		
Transient elastography	Before treatment	Before treatment
	Every 2 years thereafter	No added value after SVR
Abdominal ultrasound	6-monthly in individuals with cirrhosis and others at high risk of HCC	6-monthly in individuals with cirrhosis or with F3 fibrosis + other comorbidities

## References

[1] Collaborators GBDC. The global, regional, and national burden of cirrhosis by cause in 195 countries and territories, 1990–2017: a systematic analysis for the Global Burden of Disease Study 2017. Lancet Gastroenterol Hepatol. 2020;5(3):245–66. 31981519 10.1016/S2468-1253(19)30349-8 PMC7026710

[2] Global Burden of Disease Liver Cancer C, Akinyemiju T, Abera S, Ahmed M, Alam N, Alemayohu MA, The burden of primary liver cancer and underlying etiologies from 1990 to 2015 at the global, regional, and national level: results from the Global Burden of Disease Study 2015. JAMA Oncol. 2017;3(12):1683–91. 28983565 10.1001/jamaoncol.2017.3055 PMC5824275

[3.••] Global Hepatitis Report 2017. Geneva: World Health Organization; 2017. available at http://apps.who.int/iris/bitstream/handle/10665/255016/9789241565455-eng.pdf?sequence=1 Last accessed [10/10/2020].

[4] World Health Organization. Global Health Sector Strategy on Viral Hepatitis 2016–2021: towards ending viral hepatitis WHO/HIV/2016.06. Available at: http://www.who.int/hepatitis/strategy2016-2021/ghss-hep/en/.Accessed September 15, 2020.

[5.••] Guidelines for the prevention, care and treatment of persons with chronic hepatitis B infection. Geneva, World Health Organisation 2015. available at: http://www.who.int/hiv/pub/hepatitis/hepatitis-b-guidelines/en/ [Last accessed 8/17/2018]. 26225396

[6.••] World Health Organization (WHO). Guidelines for the care and treatment of persons diagnosed with chronic hepatitis C virus infection. Geneva, 2018 available at https://www.who.int/hepatitis/publications/hepatitis-c-guidelines-2018/en/ accessed 10/05/2020. 30307724

[7] European Association for the Study of the Liver. Electronic address eee, Clinical Practice Guidelines Panel C, representative EGB, Panel m. EASL recommendations on treatment of hepatitis C: Final update of the series(). J Hepatol. 2020. 10.1016/j.jhep.2022.10.006 36464532

[8] European Association for the Study of the Liver. Electronic address eee, European Association for the Study of the L. EASL 2017 Clinical Practice Guidelines on the management of hepatitis B virus infection. J Hepatol. 2017;67(2):370–98. 28427875 10.1016/j.jhep.2017.03.021

[9] WHO. Guidelines on Hepatitis B and C Testing. Geneva: WHO Guidelines Approved by the Guidelines Review Committee; 2017.

[10] Schweitzer A, Horn J, Mikolajczyk RT, Krause G, Ott JJ. Estimations of worldwide prevalence of chronic hepatitis B virus infection: a systematic review of data published between 1965 and 2013. Lancet. 2015;386(10003):1546–55. 26231459 10.1016/S0140-6736(15)61412-X

[11.•] Nayagam S, Conteh L, Sicuri E, Shimakawa Y, Suso P, Tamba S, Cost-effectiveness of community-based screening and treatment for chronic hepatitis B in The Gambia: an economic modelling analysis. Lancet Glob Health. 2016;4(8):e568–78. 27443782 10.1016/S2214-109X(16)30101-2

[12] Mohd Hanafiah K, Groeger J, Flaxman AD, Wiersma ST. Global epidemiology of hepatitis C virus infection: new estimates of age-specific antibody to HCV seroprevalence. Hepatology. 2013;57(4): 1333–42. 23172780 10.1002/hep.26141

[13] Grebely J, Larney S, Peacock A, Colledge S, Leung J, Hickman M, Global, regional, and country-level estimates of hepatitis C infection among people who have recently injected drugs. Addiction. 2019;114(1):150–66. 30035835 10.1111/add.14393 PMC6657799

[14] Meda N, Tuaillon E, Kania D, Tiendrebeogo A, Pisoni A, Zida S, Hepatitis B and C virus seroprevalence, Burkina Faso: a cross-sectional study. Bull World Health Organ. 2018;96(11):750–9. 30455530 10.2471/BLT.18.208603 PMC6239015

[15] Mullis CE, Laeyendecker O, Reynolds SJ, Ocama P, Quinn J, Boaz I, High frequency of false-positive hepatitis C virus enzyme-linked immunosorbent assay in Rakai, Uganda. Clin Infect Dis. 2013;57(12):1747–50. 24051866 10.1093/cid/cit602 PMC3840403

[16] Mohamed Z, Mbwambo J, Shimakawa Y, Poiteau L, Chevaliez S, Pawlotsky JM, Clinical utility of HCV core antigen detection and quantification using serum samples and dried blood spots in people who inject drugs in Dar-es-Salaam, Tanzania. J Int AIDS Soc. 2017;20(1): 21856. 28953324 10.7448/IAS.20.1.21856 PMC5964737

[17] Coffie PA, Egger M, Vinikoor MJ, Zannou M, Diero L, Patassi A, Trends in hepatitis B virus testing practices and management in HIV clinics across sub-Saharan Africa. BMC Infect Dis. 2017;17(Suppl 1):706. 29143625 10.1186/s12879-017-2768-z PMC5688463

[18] Debes JD, Kayandabila J, Pogemiller H. Knowledge of hepatitis B transmission risks among health workers in Tanzania. Am J Trop Med Hyg. 2016;94(5):1100–2. 26928835 10.4269/ajtmh.15-0797 PMC4856610

[19] Djaogol T, Coste M, Marcellin F, Jaquet A, Chabrol F, Giles-Vernick T, Prevention and care of hepatitis B in the rural region of Fatick in Senegal: a healthcare workers’ perspective using a mixed methods approach. BMC Health Serv Res. 2019;19(1):627. 31484515 10.1186/s12913-019-4416-3 PMC6727484

[20] Jaquet A, Wandeler G, Tine J, Diallo MB, Manga NM, Dia NM, Prevention and care of hepatitis b in Senegal; awareness and attitudes of medical practitioners. Am J Trop Med Hyg. 2017;97(2):389–95. 28829726 10.4269/ajtmh.17-0065 PMC5544102

[21] Mudji J, Madinga B, Horsmans Y. Seroprevalence of viral hepatitis B and C and knowledge of the hepatitis B virus among pregnant women attending prenatal care in the Democratic Republic of Congo. Am J Trop Med Hyg. 2021;104(3):1096–100. 10.4269/ajtmh.20-0804 PMC7941832 33399040

[22] Romeo R, Del Ninno E, Rumi M, Russo A, Sangiovanni A, de Franchis R, A 28-year study of the course of hepatitis Delta infection: a risk factor for cirrhosis and hepatocellular carcinoma. Gastroenterology. 2009;136(5):1629–38. 19208358 10.1053/j.gastro.2009.01.052

[23] Besombes C, Njouom R, Paireau J, Lachenal G, Texier G, Tejiokem M, The epidemiology of hepatitis delta virus infection in Cameroon. Gut. 2020;69(7):1294–300. 31907297 10.1136/gutjnl-2019-320027

[24] Stockdale AJ, Chaponda M, Beloukas A, Phillips RO, Matthews PC, Papadimitropoulos A, Prevalence of hepatitis D virus infection in sub-Saharan Africa: a systematic review and meta-analysis. Lancet Glob Health. 2017;5(10):e992–e1003. 28911765 10.1016/S2214-109X(17)30298-X PMC5599428

[25] Nerrienet E, Pouillot R, Lachenal G, Njouom R, Mfoupouendoun J, Bilong C, Hepatitis C virus infection in cameroon: a cohort-effect. J Med Virol. 2005;76(2):208–14. 15834878 10.1002/jmv.20343

[26] Yip TC, Wong GL, Chan HL, Tse YK, Lam KL, Lui GC, HBsAg seroclearance further reduces hepatocellular carcinoma risk after complete viral suppression with nucleos(t)ide analogues. J Hepatol. 2019;70(3):361–70. 30367899 10.1016/j.jhep.2018.10.014

[27.•] Chihota BV, Wandeler G, Chilengi R, Mulenga L, Chung RT, Bhattacharya D, High rates of hepatitis B virus (HBV) functional cure among human immunodeficiency virus-HBV coinfected patients on antiretroviral therapy in Zambia. J Infect Dis. 2020;221(2):218–22. 31613956 10.1093/infdis/jiz450 PMC7184905

[28] Buti M, Riveiro-Barciela M, Esteban R. Long-term safety and efficacy of nucleo(t)side analogue therapy in hepatitis B. Liver Int. 2018;38(Suppl 1):84–9. 10.1111/liv.13641 29427500

[29] Mason WS, Gill US, Litwin S, Zhou Y, Peri S, Pop O, HBV DNA Integration and clonal hepatocyte expansion in chronic hepatitis B patients considered immune tolerant. Gastroenterology. 2016;151(5):986–98 e4. 27453547 10.1053/j.gastro.2016.07.012 PMC8406433

[30.•] Yang JD, Mohamed EA, Aziz AO, Shousha HI, Hashem MB, Nabeel MM, Characteristics, management, and outcomes of patients with hepatocellular carcinoma in Africa: a multicountry observational study from the Africa Liver Cancer Consortium. Lancet Gastroenterol Hepatol. 2017;2(2):103–11. 28403980 10.1016/S2468-1253(16)30161-3

[31] Yang JD, Gyedu A, Afihene MY, Duduyemi BM, Micah E, Kingham TP, Hepatocellular carcinoma occurs at an earlier age in Africans, particularly in association with chronic hepatitis B. Am J Gastroenterol. 2015;110(11):1629–31. 26618430 10.1038/ajg.2015.289

[32] Cooper RD, Wiebe N, Smith N, Keiser P, Naicker S, Tonelli M. Systematic review and meta-analysis: renal safety of tenofovir disoproxil fumarate in HIV-infected patients. Clin Infect Dis. 2010;51(5):496–505. 20673002 10.1086/655681

[33] Surial B, Beguelin C, Chave JP, Stockle M, Boillat-Blanco N, Doco-Lecompte T, Switching from TDF to TAF in HIV/HBV co-infected individuals with renal dysfunction-a prospective cohort study. J Acquir Immune Defic Syndr. 2020;85(2):227–32. 32925387 10.1097/QAI.0000000000002429 PMC7495978

[34] Sax PE, Erlandson KM, Lake JE, McComsey GA, Orkin C, Esser S, Weight gain following initiation of antiretroviral therapy: risk factors in randomized comparative clinical trials. Clin Infect Dis. 2020;71(6):1379–89. 31606734 10.1093/cid/ciz999 PMC7486849

[35.•] Aberra H, Desalegn H, Berhe N, Mekasha B, Medhin G, Gundersen SG, The WHO guidelines for chronic hepatitis B fail to detect half of the patients in need of treatment in Ethiopia. J Hepatol. 2019;70(6):1065–71. 30929749 10.1016/j.jhep.2019.01.037

[36] Kim WR, Berg T, Asselah T, Flisiak R, Fung S, Gordon SC, Evaluation of APRI and FIB-4 scoring systems for non-invasive assessment of hepatic fibrosis in chronic hepatitis B patients. J Hepatol. 2016;64(4):773–80. 26626497 10.1016/j.jhep.2015.11.012

[37.•] Gupta N, Mbituyumuremyi A, Kabahizi J, Ntaganda F, Muvunyi CM, Shumbusho F, Treatment of chronic hepatitis C virus infection in Rwanda with ledipasvir-sofosbuvir (SHARED): a single-arm trial. Lancet Gastroenterol Hepatol. 2019;4(2):119–26. 30552056 10.1016/S2468-1253(18)30382-0

[38] Papatheodoridis GV, Sypsa V, Dalekos GN, Yurdaydin C, Van Boemmel F, Buti M, Hepatocellular carcinoma prediction beyond year 5 of oral therapy in a large cohort of Caucasian patients with chronic hepatitis B. J Hepatol. 2020;72(6):1088–96. 31981727 10.1016/j.jhep.2020.01.007

[39.•] Wandeler G, Mauron E, Atkinson A, Dufour JF, Kraus D, Reiss P, Incidence of hepatocellular carcinoma in HIV/HBV-coinfected patients on tenofovir therapy: relevance for screening strategies. J Hepatol. 2019;71(2):274–80. 30965070 10.1016/j.jhep.2019.03.032

[40] Moturi E, Tevi-Benissan C, Hagan JE, Shendale S, Mayenga D, Murokora D, Implementing a birth dose of hepatitis B vaccine in Africa: findings from assessments in 5 countries. J Immunol Sci. 2018;Suppl(5):31–40. 30931434 PMC6436721

[41] WHO/UNICEF estimates of national immunization coverageavialble at https://www.who.int/immunization/monitoring_surveillance/data/en/ accessed September 15 2020.

[42] Bassoum O, Kimura M, Tal Dia A, Lemoine M, Shimakawa Y. Coverage and timeliness of birth dose vaccination in Sub-Saharan Africa: a systematic review and meta-analysis. Vaccines (Basel). 2020;8(2):301. 10.3390/vaccines8020301 PMC7350240 32545322

[43.••] WHO. Prevention of mother-to-child transmission of hepatitis B virus: guidelines on antiviral prophylaxis in pregnancy. Geneva: WHO Guidelines Approved by the Guidelines Review Committee; 2020. 32833415

[44] Schouten EJ, Jahn A, Midiani D, Makombe SD, Mnthambala A, Chirwa Z, Prevention of mother-to-child transmission of HIV and the health-related Millennium Development Goals: time for a public health approach. Lancet. 2011;378(9787):282–4. 21763940 10.1016/S0140-6736(10)62303-3

[45] Polaris Observatory C The case for simplifying and using absolute targets for viral hepatitis elimination goals. J Viral Hepat. 2021;28(1):12–9. 32979881 10.1111/jvh.13412

[46] Wilson P, Parr JB, Jhaveri R, Meshnick SR. Call to action: prevention of mother-to-child transmission of hepatitis B in Africa. J Infect Dis. 2018;217(8):1180–3. 29351639 10.1093/infdis/jiy028 PMC6279162

[47] Ford N, Singh K, Cooke GS, Mills EJ, von Schoen-Angerer T, Kamarulzaman A, Expanding access to treatment for hepatitis C in resource-limited settings: lessons from HIV/AIDS. Clin Infect Dis. 2012;54(10):1465–72. 22431808 10.1093/cid/cis227

